# Proposed Medical Convention in Columbus, Ohio

**Published:** 1834

**Authors:** 


### V.—Proposed Medical Convention in Columbus, Ohio.

We have received from Dr. W. McClay Awl of Columbus, a printed paper, calling a convention of the physicians of Ohio, in January next. We insert it below, and beg leave to commend it to the attention of our Ohio readers. As the objects which Dr. Awl and his friends propose to advance, by the projected convention, are of deep interest to the profession and to society, we sincerely hope it will be generally attended. We have applied the word profession, to the physicians of Ohio, but it is scarcely admissible thus to designate them. They are not united into a profession. With a few exceptions they are strangers to each other; without common objects, common sympathies, or common aspirations. The proposed convention, should the medical gentlemen of the state, generally, appear in it, cannot fail to make them much better acquainted with each other, and prepare them for concert of action, in whatever may hereafter call for it. The following is the printed circular.

TO ALL THE PRACTITIONERS OF MEDICINE AND SURGERY, IN THE STATE OF OHIO.

The undersigned, uniting in sentiment and feeling with that portion of the Profession who view, with pain, the great depression of character—want of harmony and concentration of useful action, which unhappily prevail in the Medical Science—acknowedging, also, a proper responsibility for the advancement of correct principles, the promotion of public benevolence, and the common welfare of society,—are induced most respectfully to recommend and consent to support a call for the assemblage of a General Medical Convention, to be holden in the city of Columbus, on Monday, the 5th of January, A. D. 1835.

The grand design is to organize for practical utility, the whole scientific medical power of the State. All regular scientific Practitioners of Medicine and Surgery, either of city, village, or country, who are disposed to advance the honor and dignity of the profession;—every one who has a heart in the cause of science, and is ready to unite with the great and good of the age, in elevating the moral and scientific character and talent of the great and extending West, is cordially invited, and expected to come and record his name in this Convention.

The regulation of professional etiquette—

The construction of independent Medical Societies—

The support of a periodical Journal of Practical Medicine—

The erection and location of public Asylums, for the reception of Lunatics and the instruction of the Blind—

The promotion of the Temperance cause—

The regulation of Vaccination—

The convenient supply of the Leech—and many others subjects, will perhaps claim the attention of the Convention. But as the whole proceeding should be an independent and voluntary offering for the common good, all are expected to be unpledged, and none should come entirely unprepaired.

